# Morphological differences in the calcaneus among extant great apes investigated by three-dimensional geometric morphometrics

**DOI:** 10.1038/s41598-021-99942-1

**Published:** 2021-10-22

**Authors:** Shuhei Nozaki, Hideki Amano, Motoharu Oishi, Naomichi Ogihara

**Affiliations:** 1 Laboratory of Human Evolutionary Biomechanics, Department of Biological Sciences, Graduate School of Science, The University of Tokyo, 7-3-1 Hongo, Bunkyo, Tokyo, 113-0033 Japan; 2 Laboratory of Anatomy, School of Veterinary Medicine, Azabu University, Kanagawa, 252-0206 Japan

**Keywords:** Evolution, Anthropology

## Abstract

Investigating the morphological differences of the calcaneus in humans and great apes is crucial for reconstructing locomotor repertories of fossil hominins. However, morphological variations in the calcaneus of the great apes (chimpanzees, gorillas, and orangutans) have not been sufficiently studied. This study aims to clarify variations in calcaneal morphology among great apes based on three-dimensional geometric morphometrics. A total of 556 landmarks and semilandmarks were placed on the calcaneal surface to calculate the principal components of shape variations among specimens. Clear interspecific differences in calcaneal morphology were extracted, corresponding to the degree of arboreality of the three species. The most arboreal orangutans possessed comparatively more slender calcaneal tuberosity and deeper pivot region of the cuboid articular surface than chimpanzees and gorillas. However, the most terrestrial gorillas exhibited longer lever arm of the triceps surae muscle, larger peroneal trochlea, more concave plantar surface, more inverted calcaneal tuberosity, more everted cuboid articular surface, and more prominent plantar process than the orangutans and chimpanzees. These interspecific differences possibly reflect the functional adaptation of the calcaneus to locomotor behavior in great apes. Such information might be useful for inferring foot functions and reconstructing the locomotion of fossil hominoids and hominids.

## Introduction

The human foot possesses a calcaneus, the tuberosity of which points posteriorly and inferiorly, allowing a prominent heel strike during bipedal walking^1,2^. African great apes also have plantigrade feet and can walk with a heel strike^1,3–7^, but heel and midfoot often contact the ground at the same time^6^, and the heel strike in African great apes is not as prominent as that in humans. Old world monkeys, such as Japanese macaques, usually touch down with the fore- and midfoot but do not walk with a heel strike^1,8,9^.

Reflecting these differences in foot–ground contact between humans and African great apes during locomotion, the calcaneus morphology differs largely among species. Previous studies noted that: (1) the human calcaneus is more robust, mediolaterally wider, and longitudinally straighter than that of the African apes^1,2,10^; (2) the human calcaneus possesses a large, robust tuberosity and plantarly located lateral plantar process^2,11^; (3) the human calcaneus possesses a peroneal trochlea that is smaller than that of the African apes^12,13^; (4) the calcaneocuboid articular surface is more acutely angled with respect to the longitudinal axis of the calcaneus in humans than in African apes in lateral view due to the longitudinally arched structure of the human foot^14^; (5) the calcaneocuboid articular surface is more flat and asymmetrical, constricting rotatory movement at the joint in humans than in African apes^15,16^; and (6) the posterior talar facet is flatter in the human calcaneus than in that of the African apes, reflecting more constricted subtalar joint in humans^17^. These morphological differences distinguishing the human calcaneus from those of African apes are considered as morphological adaptations to obligatory bipedal locomotion, and are used to reconstruct the locomotor repertories of early hominins to understand the origin and evolution of habitual bipedal locomotion in the human lineage^1,2,11,17,18^.

These studies focused on documenting the calcaneus morphological variation between humans and African apes, assuming it to be relatively minor among chimpanzees and gorillas. However, there exist calcaneal morphological differences among great apes that may be correlated with differences in their locomotor behavior^2,19^. Great apes (African great apes plus orangutans) utilize a versatile locomotor repertoire such as suspensory locomotion (brachiation), vertical climbing, quadrumanous climbing, and terrestrial quadrupedal locomotion (knuckle walking)^20–25^. Therefore, a detailed analysis of the morphological variations of the calcaneus among great apes would provide opportunities to clarify the form-function relationships in the calcaneus critical for predicting the foot function of fossil hominoids and hominids.

Using three-dimensional (3D) geometric morphometrics, DeSilva et al.^26^ conducted a morphological analysis of hominin fossil calcanei, including human and great ape samples, based on a relatively small number of anatomical landmarks (20), and no explicit comparisons among the great ape calcanei were made, as the aim was to identify the overall morphological affinity of the fossil calcanei in the calcanei of humans and great apes^18,26^. Harcourt-Smith^27^ analyzed the patterns of morphological variations of human and great ape calcanei based on 20 landmarks, but only crude comparisons were possible because of the small number of landmarks. Harper et al.^28^ has recently clarified the calcaneal morphological variations among the three subspecies of gorilla taxa with differences in the degree of arboreality based on geometric morphometrics of approximately a thousand landmarks and demonstrated that the calcaneus is anteroposteriorly more elongated and possesses more concave cuboid and flatter posterior talar articular surfaces. However, only these studies have explored morphological variations among great apes calcanei using geometric morphometrics. Therefore, this present study aimed to quantify detailed variations in calcaneal morphology among great apes based on 3D morphometrics using a sufficiently large number of landmarks (> 500). Specifically, we investigated whether the differences in the calcaneal morphology, particularly the differences in the shapes of the calcaneal tuberosity and the articular surfaces of the calcaneus among great apes possibly corresponded to the differences in their locomotor behaviors and degree of arboreality.

## Materials and methods

### Sample

Computed tomography (CT) scans of calcanei from 20 chimpanzees (10 *P. troglodytes troglodytes*, 5 *P. troglodytes schweinfurthii*, 4 *P. troglodytes verus*, and 1 *P. troglodytes hybrid*), 20 gorillas (14 *G. gorilla gorilla* and 6 *G. beringei beringei*), and 20 orangutans (19 *P. pygmaeus* and 1 *P. abelii*) were used for the analysis. Specimens of seven chimpanzees, five gorillas, and four orangutans were captive cadaver feet donated to the Primate Research Institute, Kyoto University, Japan. Wild specimens of 10 chimpanzees, 15 gorillas, and 14 orangutans were obtained from MorphoSource (https://www.morphosource.org, Media ID: 101819; 101887; 101912; 101994; 102264; 102393; 102599; 102710; 102915; 103083; 2136; 2139; 2142; 51600; 51836; 51869; 84188; 84195; 85860; 86050; 86054; 101836; 102164; 102220; 103649; 2148; 2344; 102540; 102672; 102822; 102921; 102966; 103119; 103220; 103477; 103526; 103580; 103586; 103645). Three dry bone specimens of chimpanzees were wild individuals from the Mahale Mountain National Park, Tanzania. Two orangutans dry bone specimens were collected at the Laboratory of Physical Anthropology, Kyoto University. In addition, calcanei from 10 humans and 7 Japanese macaques (*Macaca fuscata*) were also included in this analysis as outgroups. The human specimens were dry bone specimens housed at the University Museum, University of Tokyo. The Japanese macaque samples were captive dry bone specimens housed at the Laboratory of Physical Anthropology, Kyoto University, except for one cadaver specimen studied by Ogihara et al.^29^. All samples were adults and were free of obvious pathology. All calcanei were segmented from the CT scans, and 3D bone models of the calcanei were generated in Mimics 22.0 (Materialise Inc., Leuven, Belgium). The left calcanei were mirrored and analyzed as right-sided specimens. Pixel size and slice interval of the CT scans were < 1 mm for great apes, and < 0.2 mm for humans and Japanese macaques.

### Three-dimensional geometric morphometrics

**Figure 1:**
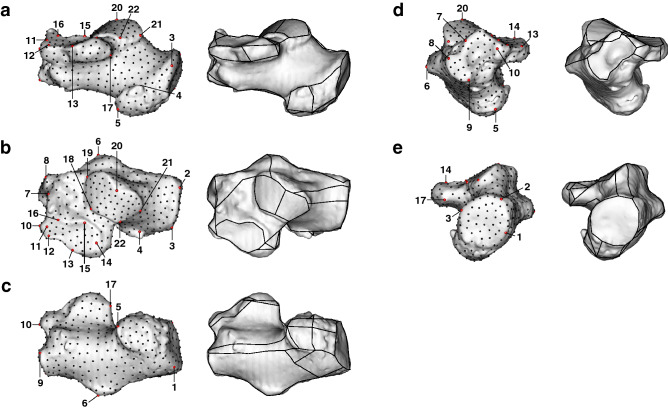
Landmarks used in the present study. Twenty-two anatomical landmarks (red) and 534 semi-landmarks (black) were defined on a representative chimpanzee calcaneal specimen chosen as a template. Wireframes connecting primary landmarks were drawn to visualize shape variations. (**a**) Medial view, (**b**) Superior view, (**c**) Inferior view, (**d**) Anterior view, (**e**) Posterior view.

Calcaneal shape variations were analyzed using sliding-semilandmark-based 3D geometric morphometrics. A total of 22 anatomical landmarks were digitized on the 3D surface of the calcaneus (Fig. 1, Table 1). In addition, we manually defined 534 sliding semilandmarks on one chimpanzee specimen chosen as a template specimen (Fig. 1), to fully represent the entire calcaneal bone and articular surfaces. The semilandmark configuration of the template was mapped to all other specimens in the sample using a thin-plate spline function^30^ and subsequently allowed to slide to minimize the bending energy between each specimen and the template^31,32^. The shape variation of the calcanei among species were analyzed using geometric morphometrics^33–35^. The coordinates of the 556 landmarks were normalized by centroid size and registered using the generalized Procrustes method^36–38^ to remove variance associated with size, translation, and orientation. Principal component analysis (PCA) was then conducted using the variance–covariance matrix of the Procrustes residuals to obtain the principal components (PCs) of shape variations among the specimens. In this study, we analyzed the morphological variations of the calcanei using PCA in two ways: (1) a whole-sample analysis using all samples, including human and macaque samples, to grasp a broader picture of the shape variations of the great ape calcaneus; and (2) an ape-only analysis using only chimpanzee, gorilla, and orangutan samples to illustrate the differences in the calcaneal morphology among the three. The calcaneal shape variations along the PCs were represented by warping either the wireframe connecting the primary landmarks (Fig. 1) or the surface model of the template calcaneus. The mean calcaneal shapes of the chimpanzee, gorilla and orangutan were also calculated and presented in the same manner for interspecific comparisons (See Supplementary Information).

**Table 1:** Description of landmarks used in this study.

Number	Description
1	Most lateral point of the calcaneal tuberosity
2	Most superior point of the calcaneal tuberosity
3	Most medial point of the calcaneal tuberosity
4	Midpoint between point 3 and 5 on the medial edge of the calcaneal tuberosity
5	Most anterior point of the medial plantar process
6	Apex of the peroneal trochlea
7	Most lateral point of the articular surface for cuboid
8	Most inferolateral point of the articular surface for cuboid
9	Most inferomedial point of the articular surface for cuboid
10	Most superomedial point of the articular surface for cuboid
11	Most anterior point of the anterior talar articular surface
12	Most medial point of the anterior talar articular surface
13	Most medial point of the middle talar articular surface
14	Most posterior point of the middle talar articular surface
15	Most lateral point of the middle talar articular surface
16	Most lateral point of the anterior talar articular surface
17	Most posteroinferior point of the sustentaculum tali
18	Most anterolateral point of the posterior talar articular surface
19	Most lateral point of the posterior talar articular surface
20	Most posterior point of the posterior talar articular surface
21	Most medial point of the posterior talar articular surface
22	Most anteromedial point of the posterior talar articular surface

### Statistical analyses

Inter-specific shape differences were analyzed for the PCs using analysis of variance (ANOVA). If the ANOVA was significant, a post-hoc Tukey’s HSD test was performed. The statistical significance level was set at *P* < 0.05. The Kruskal–Wallis test with a post-hoc Wilcoxon rank sum test for multiple comparisons, followed by Bonferroni correction with the adjusted *P*-value set at *P* < 0.005 (0.05/10), was used if normality or homogeneity of variance was violated. Data processing and analyses were implemented in R software, version 3.5.2^39^, using the R package ‘geomorph’ and ‘Morpho’.

The present study included both wild and captive specimens for geometric morphometric analysis. To investigate possible differences in the calcaneal morphology between the wild and captive specimens of the same species, we tested if the mean PC scores were significantly different between the wild and captive specimens in chimpanzees, gorillas and orangutans using two-tailed *t*-test or Wilcoxon rank-sum test.

## Results

### Whole-sample analysis

**Figure 2:**
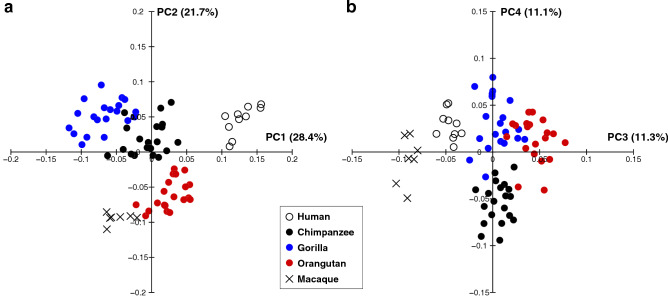
The results of the whole-sample analysis represented as scatter plots of the principal component PC1 versus PC2 (**a**) and PC3 versus PC4 (**b**). The percentage of variance explained by each PC score is shown in parentheses.

The results of the whole-sample analysis are presented in Fig. 2 as scatter diagrams of PC1 versus PC2 and PC3 versus PC4. Based on a threshold of 5% variance explained, the first four PCs were considered dominant in the whole-sample analysis. The first four PCs accounted for 72.5% of the total variance. ANOVA and the Kruskal–Wallis test indicated that there were significant differences in PC1 (*F* = 121.3, *P* < 0.0001), PC2 (*x*^2^ = 60.3, *P* < 0.0001), PC3 (*F* = 164.5, *P* < 0.0001), and PC4 (*F* = 39.0, *P* < 0.0001) among the species.

**Figure 3:**
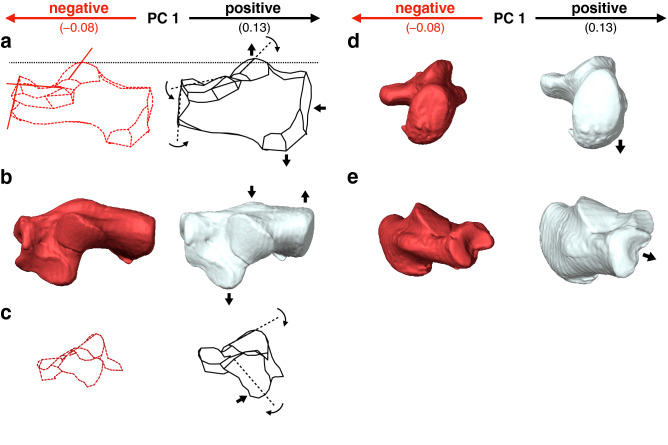
Shape variations represented by PC1 in the whole-sample analysis. Shape variations are visualized using the wireframe connecting primary landmarks and the template surface deformed using a thin-plate spline function. Black line and white surface: PC1 = 0.13. Red line and red surface: PC1 = − 0.08. Black arrows indicate characteristics of the positive score. The medial (**a**), superior (**b**), posterior views of the anterior (**c**) and posterior (**d**) parts, and anterolateral (**e**) views were presented.

Along PC1, humans were separated (Fig. 2a) and exhibited significantly higher PC1 scores than those of great apes and macaques (Table 2). Humans possessed a relatively larger calcaneal tuberosity (Fig. 3a, b) with plantarly located lateral plantar process (Fig. 3d), relatively smaller peroneal trochlea (Fig. 3b), and flatter and more plantarly oriented cuboid articular surface with respect to its longitudinal axis (Fig. 3a and e), as indicated previously (see “Introduction”). Also, the cuboid articular surface in humans was observed to be more twisted in the inverting direction (Fig. 3c) and faced more medially than those in great apes (Fig. 3a). Furthermore, in humans, the sagittal angle between the anterior-middle and posterior articular surfaces was larger (Fig. 3a), and the posterior articular surface was less tilted medially in the coronal plane (Fig. 3c) than in great apes. Moreover, the outline of the cuboid articular surface in humans was wedged, but it was round in the great apes (Fig. 3c).

**Table 2:** *P*-value of post-hoc tests for multiple comparisons in whole-sample analysis for PC1 and PC2.

PC1 and PC2	Human	Chimpanzee	Gorilla	Orangutan	Macaque
Human		** < 0.0001**	**< 0.0001**	**< 0.0001**	**< 0.0001**
Chimpanzee	0.006		**< 0.0001**	**0.0008**	**0.001**
Gorilla	0.619	**0.0001**		**< 0.0001**	0.351
Orangutan	** < 0.0001**	** < 0.0001**	**< 0.0001**		**< 0.0001**
Macaque	**0.0001**	** < 0.0001**	**< 0.0001**	**< 0.0001**	

The right-upper cells represent the *P*-value for PC1, and the left-lower cells represent PC2.

*P*-values < 0.05 are in bold for PC1, and *P*-values < 0.005 (0.05/10) are in bold for PC2 to indicate significant differences.

**Figure 4:**
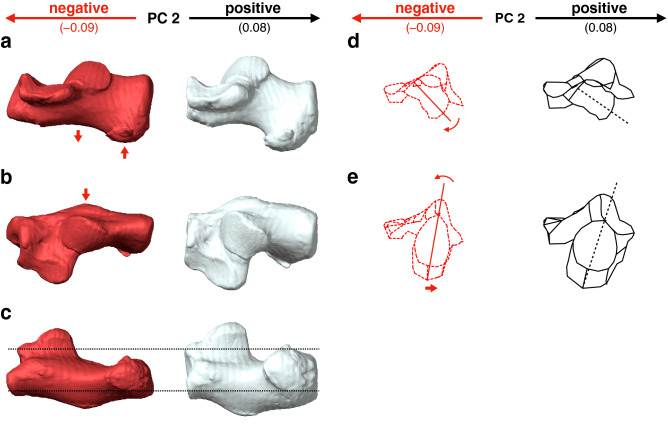
Shape variations represented by PC2 in the whole-sample analysis. Shape variations are visualized using the wireframe connecting primary landmarks and the template surface deformed using a thin-plate spline function. Black line and white surface: PC2 = 0.08. Red line and red surface: PC2 = − 0.09. Red arrows indicate characteristics of the negative score. The medial (**a**), superior (**b**), inferior (**c**), and posterior views of the anterior (**d**) and posterior (**e**) parts were presented.

Along PC2, macaques were separated and exhibited significantly smaller PC2 scores than the other four species (Fig. 2a, Table 2). They had a less inferiorly prominent and mediolaterally narrower plantar process (Fig. 4a, c), more convex plantar surface of the calcaneus (Fig. 4a), smaller peroneal trochlea (Fig. 4b), less inverted calcaneal tuberosity with more laterally shifted medial plantar process (Fig. 4e), and less everted cuboid articular surface (Fig. 4d) than great apes and humans.

The PC3 and PC4 scores were also significantly different among the five species (Fig. 2b, Table 3), but since the present study focused on the calcaneal shape variations within the three great ape species, these scores were not considered.

**Table 3:** *P*-value of post-hoc tests for multiple comparisons in whole-sample analysis for PC3 and PC4.

PC3 and PC4	Human	Chimpanzee	Gorilla	Orangutan	Macaque
Human		** < 0.0001**	** < 0.0001**	**< 0.0001**	**< 0.0001**
Chimpanzee	** < 0.0001**		0.996	**< 0.0001**	**< 0.0001**
Gorilla	1.000	**< 0.0001**		**< 0.0001**	**< 0.0001**
Orangutan	0.468	**< 0.0001**	0.183		** < 0.0001**
Macaque	**0.020**	**0.0004**	**0.004**	0.249	

The right-upper cells represent the *P*-value for PC3 and the left-lower cells represent PC4.

*P*-values < 0.05 are in bold for PC3 and PC4 to indicate significant differences.

### Ape-only analysis

**Figure 5:**
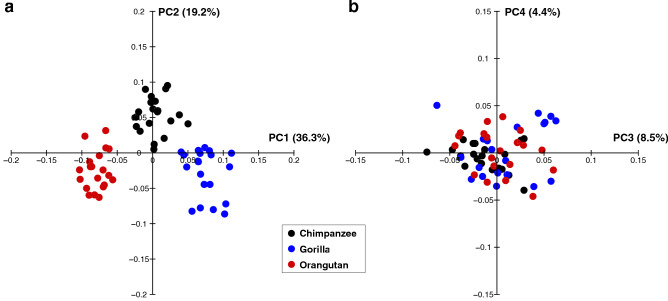
The results of the ape-only analysis represented as scatter plots of the principal component (PC) 1 versus PC2 (**a**) and PC3 versus PC4 (**b**). The percentage of variance explained by each PC score is shown in parentheses.

The results of the ape-only analysis are presented in Fig. 5 as scatter diagrams of PC1 versus PC2 and PC3 versus PC4. Based on a threshold of 5% variance explained, the first three PCs were considered dominant in the ape-only analysis. The first three PCs accounted for 64.0% of the total variance. We confirmed that there were no statistically significant differences in the PC scores between the wild and captive specimens, except for the PC3 score of orangutans (Supplementary Fig. 1 and Supplementary Table), suggesting that the use of captive specimens has only minor effect on the results of the present analysis.

**Figure 6:**
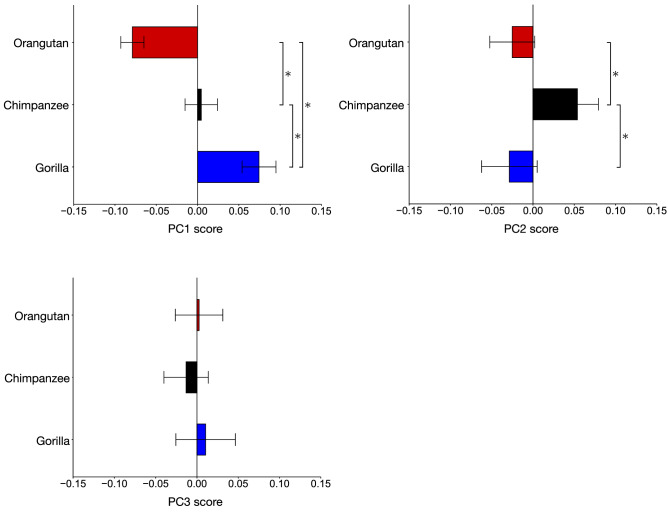
Interspecific differences in the mean principal component scores in the ape-only analysis. The asterisk indicates that there is significant difference in the PC scores among species (*P* < 0.05).

The PC1 versus PC2 plot demonstrated that plots of chimpanzees, gorillas, and orangutans were clearly separated from one another. The ANOVA indicated that there were significant differences in PC1 (*F* = 353.2, *P* < 0.0001) and PC2 (*x*^2^ = 37.4, *P* < 0.0001) scores among these species. Post-hoc tests showed that PC1 was significantly different among the three groups (Figs. 5a, 6). In particular, the mean PC1 score was arranged in the order of orangutans, chimpanzees, and gorillas in ascending order.

**Figure 7:**
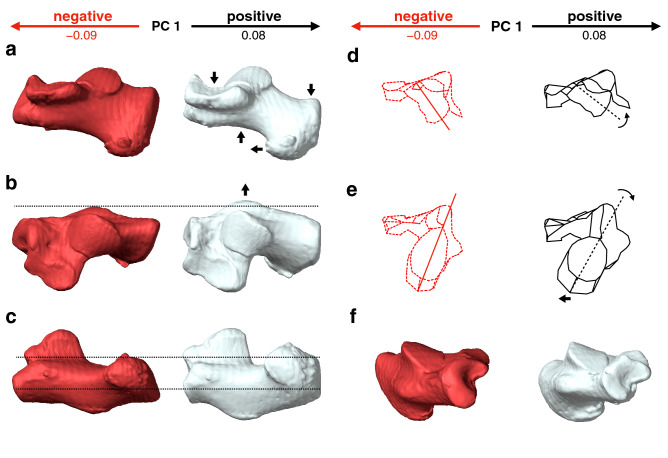
Shape variations represented by PC1 in the ape-only analysis. Shape variations are visualized using the wireframe connecting primary landmarks and the template surface deformed using a thin-plate spline function. Black line and white surface: PC1 = 0.08. Red line and red surface: PC1 = − 0.09. Black arrows indicate characteristics of the positive score. The medial (**a**), superior (**b**), inferior (**c**), posterior views of anterior (**d**) and posterior (**e**) parts, and anterolateral (**f**) views were presented.

With increasing PC1, the height of the calcaneal tuberosity decreased while its mediolateral width increased (Fig. 7a, c), indicating robust calcaneal tuberosity in African apes. The plantar surface of the calcaneus was more concave (Fig. 7a), the heel process was more prominent (Fig. 7a), and the peroneal trochlea became larger (Fig. 7b) with increasing PC1. In the coronal plane, the calcaneal tuberosity was more twisted in the inverting direction, and the medial plantar process was consequently shifted medially (Fig. 7e). In contrast, the cuboid articular surface was more twisted in the everting direction with increasing PC1 (Fig. 7d). Furthermore, the anterior-middle and posterior articular surfaces were located closer to each other, but the distance between the posterior articular surface and the calcaneal tuberosity increased with increasing PC1 (Fig. 7b). The medial and lateral cuboid articular surfaces were flatter with increasing PC1 (Fig. 7f), but they were more concave and convex with decreasing PC1 (Fig. 7c).

**Figure 8:**
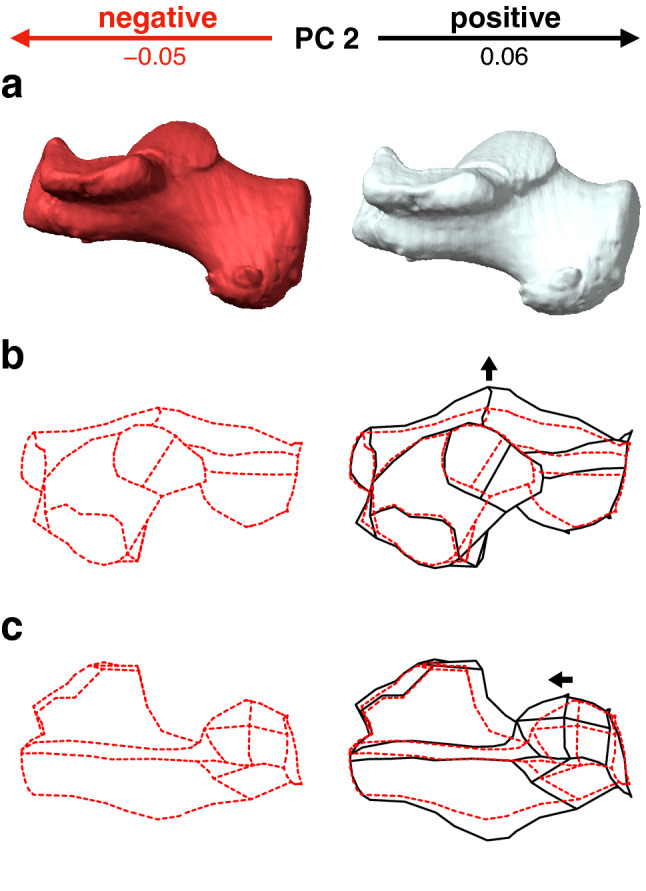
Shape variations represented by PC2 in the ape-only analysis. Shape variations are visualized using the wireframe connecting primary landmarks and the template surface deformed using a thin-plate spline function. Black line and white surface: PC2 = 0.06. Red line and red surface: PC2 = − 0.05. Black arrows indicate characteristics of the positive score. The medial (**a**), superior (**b**), and inferior (**c**) views were presented.

PC2 was significantly larger in chimpanzees than in orangutans and gorillas. With increasing PC2, the length of the posterior articular surface increased (Fig. 8b), the heel process was located more anteriorly (Fig. 8a, c), the peroneal trochlea was larger, and the calcaneal body was wider (Fig. 8b). However, the shape variations represented by PC2 were similar to those extracted in PC1, but the contribution was much smaller than that of PC1. Therefore, shape variations along PC2 will not be discussed further.

## Discussion

Although the calcaneal shape of the extant great apes is generally similar to one another (Fig. 2a), the present study demonstrated that there is a clear, statistically significant shape difference in the calcaneus among the extant great apes (Fig. 5a). The extracted shape variation, represented by PC1 of the ape-only analysis, possibly corresponds to locomotor behaviors and the degree of arboreality of the three species; the fundamental quadrumanous climbers orangutans are the most arboreal among the extant great apes having the lowest mean PC1 score, the gorillas are the most terrestrial great apes with the highest mean PC1 score, and the chimpanzees fell between the two. The extracted shape variations along PC1 include the tendency of the medial and lateral pivot regions of the cuboid facet to be more deeply concave and convex, respectively, with decreasing PC1 (in orangutans) but flatter with increasing PC1 (in gorillas). The morphology of the cuboid facet pivot region is linked to greater and lesser midfoot joint mobility, which is considered to reflect an adaptation for arboreal and terrestrial locomotion, respectively^1,28^.

The shape variation along PC1 also indicated that the distances between the anterior-middle and posterior articular surfaces decreased, but the distance between the posterior articular surface and the calcaneal tuberosity increased with increasing PC1, and vice versa (Fig. 7b) as previously suggested by Harcourt-Smith^27^. Therefore, the longer moment arm length of the triceps surae muscle (the Achilles tendon) is related to more terrestrial behaviors. In addition, the peroneal tubercle was more laterally protrudent with increasing PC1 and vice versa (Fig. 7b), suggesting that the size of the peroneal tubercle is also related to the degree of terrestriality in great apes. It must be noted, however, that the longer moment arm of the triceps surae muscle should be advantageous during the propulsive phase not only in terrestrial quadrupedal locomotion but also in arboreal vertical climbing. Furthermore, the large robust peroneal tubercle reportedly linked to a large force applied by the peroneal muscles^13^, should also be advantageous to evert the foot during both terrestrial and arboreal locomotion. Therefore, these morphological differences may not represent the difference in the degree of terrestriality or arboreality in great apes. Therefore, care should be taken when making inferences about the locomotor behaviors of fossil hominids based on the morphology of the peroneal tubercle.

The present study also demonstrated that the calcaneal tuberosity was more inverted with respect to the body of the calcaneus with increasing PC1 and vice versa (Fig. 7e), indicating that the calcaneal tuberosity is inverted and everted in relatively terrestrial and arboreal great apes, respectively. The feet of great apes are highly inverted to facilitate positioning of the sole of the foot against the vertical substrate during climbing as an adaptation to arboreal life^40^. However, the present study indicates the orientation of the calcaneal tuberosity did not necessarily correspond to the inversion/eversion of the foot with respect to the shank in great apes. In addition, the cuboid facet was more everted with increasing PC1, indicating that the more terrestrial species possessed more everted cuboid facets, possibly facilitating firm contact of the forefoot and digits of the inverted hindfoot to the ground during terrestrial locomotion. Therefore, the greater inversion of the calcaneal tuberosity and eversion of the cuboid facet might be related to terrestrial locomotion, and this morphological characteristic can possibly be better utilized to infer the locomotor behavior of the fossil hominids. However, notably the calcaneal tuberosity and the cuboid facet were less inverted and everted, respectively, in the calcaneus of habitually terrestrial humans (Fig. 3c and d).

The present study observed that the plantar process of the calcaneal tuberosity was more inferiorly projected and the plantar surface of the calcaneus was more concave (Fig. 7a) with increasing PC1, possibly indicating that these morphological characteristics were related to the degree of terrestrial locomotion. However, Sarmiento^41^ previously proposed that this morphological feature is an adaptation to a strong hook-like grasp for hanging and tree climbing because it provides a broader insertion site for the flexor digitorum brevis muscle (FDB). If so, the plantar process in orangutans should have been the largest in great apes, but the present study indicated the opposite. The reason behind this discrepancy is currently obscure, but a recent study indicated that the FDB greatly contributed to the generation of an effective propulsive force during push-off in human walking^42^. In addition, the plantar process is the attachment site of the plantar aponeurosis, which is mostly well developed in gorillas among the extant great apes^43^ as an adaptation to terrestrial locomotion^44^. Sarmiento^41^ indicated that the concave plantar surface of the calcaneus with the developed plantar process in humans and gorillas can be seen as a specialization retained from a common ancestor that engaged in foot hanging and tree climbing, but our study suggests that these could be regarded as an adaptation to terrestrial locomotion in extant great apes.

In addition, the present detailed geometric morphometric study revealed that the angle between the anterior-middle and posterior articular surfaces was larger in the sagittal plane (Fig. 3a), and the posterior articular surface was less tilted medially in the coronal plane (Fig. 3c) than in the great apes, indicating that the overall talar articular surface was more planar in the human calcaneus. The flattening of the talar articular surfaces in humans possibly facilitates the medial translation and internal rotation of the talus on the calcaneus and hence the internal rotation of the tibia following calcaneal eversion after the heel strike during walking^45–48^. This morphological feature of the human calcaneus may enhance the coupling motion of the calcaneus and tibia during walking, which is structurally embedded in the human foot as an adaptation to obligatory bipedal locomotion^49^.

In conclusion, the present study demonstrated that there are interspecific differences in the calcaneal shape among chimpanzees, gorillas, and orangutans based on 3D geometric morphometrics, and suggested that the extracted pattern of calcaneal shape variation possibly corresponds to the differences in the degree of terrestriality/arboreality among the three species. A detailed understanding of the morphological variations of the calcaneus among great apes would provide valuable opportunities to clarify the form-function relationships in the calcaneus, which is critical for predicting the foot function of fossil hominoids and hominids.

## Supplementary Information

Supplementary Information.

## Data Availability

The data that support the findings of this study are available from the corresponding authors upon reasonable request.
