# Supplementary material for: Morphological differences in the calcaneus among extant great apes investigated by three-dimensional geometric morphometrics
Source: Sci Rep. 2021 Oct 22;11:20889. doi: 10.1038/s41598-021-99942-1 (PMC8536676; doi:10.1038/s41598-021-99942-1)
Supplement: Supplementary file 1 — Supplementary Information. [file 41598_2021_99942_MOESM1_ESM.pdf]

Supplementary Information

Morphological differences in the calcaneus among extant great apes investigated by three-dimensional geometric morphometrics

Shuhei Nozaki[1*], Hideki Amano[1], Motoharu Oishi[2], and Naomichi Ogihara[1*]

[1]Laboratory of Human Evolutionary Biomechanics, Department of Biological Sciences, Graduate School of Science, The University of Tokyo, Tokyo, 113-0033, Japan.

[2]Laboratory of Anatomy, School of Veterinary Medicine, Azabu University, Kanagawa, 252-0206, Japan.

Supplementary Table. *P*-value of statistical tests (two-tailed *t*-test or Wilcoxon rank-sum test) to compare between the wild and captive specimens in ape-only analysis

species	PC1	PC2	PC3
Chimpanzee	0.332	0.438	0.238
Gorilla	0.080	0.395	0.671
Orangutan	0.382	0.207	**0.002**

P-value < 0.017 (0.05/3) is in bold to indicate significant difference.

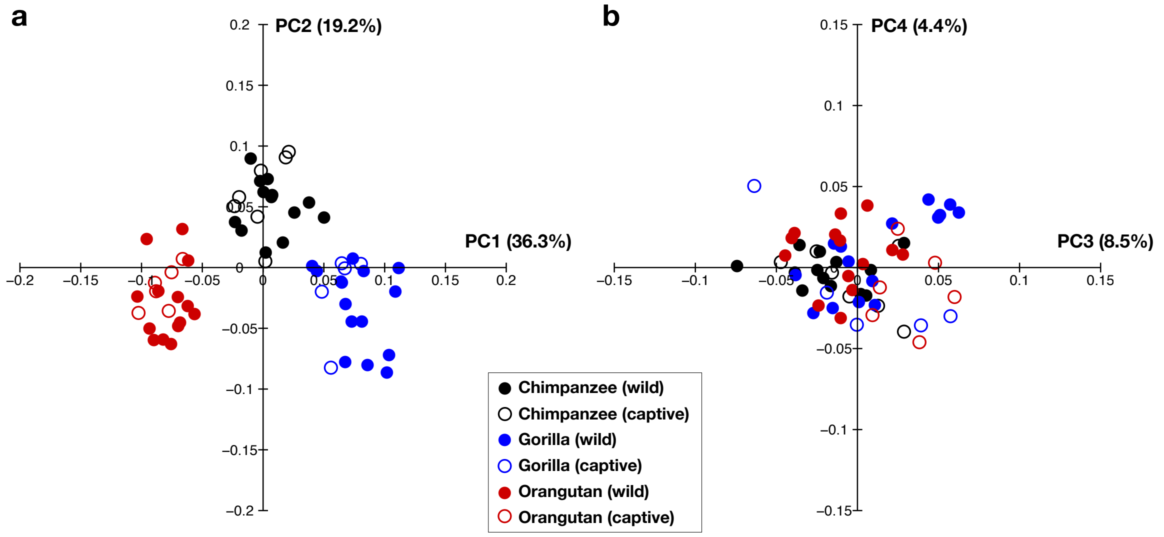

Supplementary Figure 1. Scatter plots of the PC1 versus PC2 (**a**) and PC3 versus PC4 (**b**) in ape-only analysis (wild =solid circle, captive = open circle). The percentage of variance explained by each PC score is shown in parentheses.

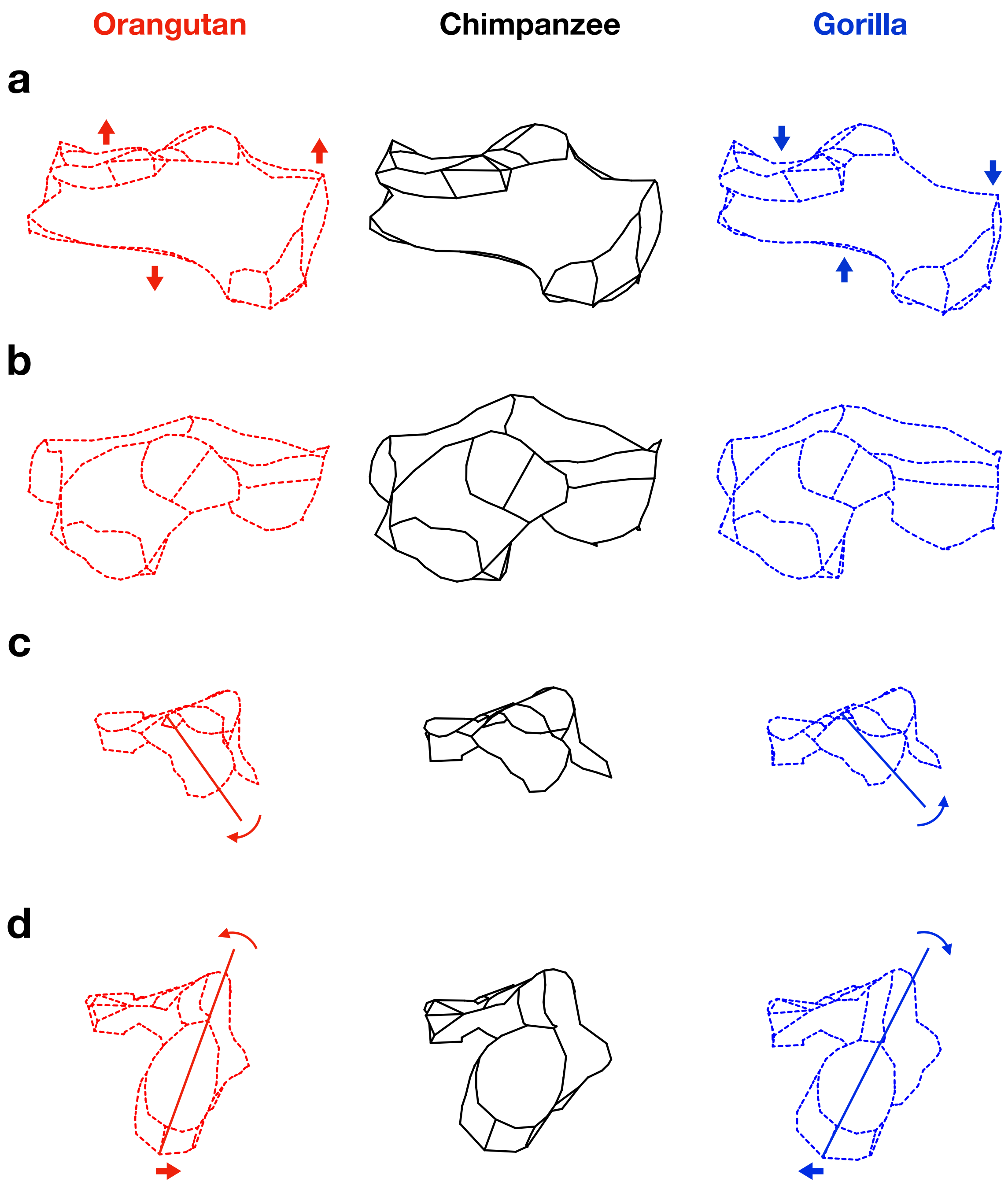

Supplementary Figure 2. Mean calcaneal shapes of the chimpanzee, gorilla and orangutan. Shapes are visualized using the wireframe connecting landmarks. Red line (orangutan): PC1 = −0.08, PC2 = −0.03. Black line (chimpanzee): PC1 = 0.004, PC2 =0.05. Blue line (gorilla): PC1 = 0.07, PC2 =−0.03. The medial view (**a**), superior view (**b**), posterior view of the anterior part (**c**), and posterior view (**d**) were presented.
